# Reliability and validity of digital health metrics for assessing arm and hand impairments in an ataxic disorder

**DOI:** 10.1002/acn3.51493

**Published:** 2022-02-27

**Authors:** Christoph M. Kanzler, Isabelle Lessard, Roger Gassert, Bernard Brais, Cynthia Gagnon, Olivier Lambercy

**Affiliations:** ^1^ Rehabilitation Engineering Laboratory, Department of Health Sciences and Technology Institute of Robotics and Intelligent Systems, ETH Zurich Zurich Switzerland; ^2^ Future Health Technologies Singapore‐ETH Centre, Campus for Research Excellence And Technological Enterprise (CREATE) Singapore; ^3^ Groupe de Recherche Interdisciplinaire sur les Maladies Neuromusculaires (GRIMN) Centre Intégré Universitaire de Santé et de Services Sociaux du Saguenay–Lac‐St‐Jean Saguenay Quebec Canada; ^4^ The Montreal Neurological Institute and Hospital McGill University Montreal Quebec Canada; ^5^ Faculty of Medicine and Health Sciences Université de Sherbrooke Sherbrooke Quebec Canada

## Abstract

**Objectives:**

Autosomal recessive spastic ataxia of Charlevoix‐Saguenay (ARSACS) is the second most frequent recessive ataxia and commonly features reduced upper limb coordination. Sensitive outcome measures of upper limb coordination are essential to track disease progression and the effect of interventions. However, available clinical assessments are insufficient to capture behavioral variability and detailed aspects of motor control. While digital health metrics extracted from technology‐aided assessments promise more fine‐grained outcome measures, these have not been validated in ARSACS. Thus, the aim was to document the metrological properties of metrics from a technology‐aided assessment of arm and hand function in ARSACS.

**Methods:**

We relied on the Virtual Peg Insertion Test (VPIT) and used a previously established core set of 10 digital health metrics describing upper limb movement and grip force patterns during a pick‐and‐place task. We evaluated reliability, measurement error, and learning effects in 23 participants with ARSACS performing three repeated assessment sessions. In addition, we documented concurrent validity in 57 participants with ARSACS performing one session.

**Results:**

Eight metrics had excellent test–retest reliability (intraclass correlation coefficient 0.89 ± 0.08), five low measurement error (smallest real difference % 25.4 ± 5.7), and none strong learning effects (systematic change *η* −0.11 ± 2.5). Significant correlations (*ρ* 0.39 ± 0.13) with clinical scales describing gross and fine dexterity and lower limb coordination were observed.

**Interpretation:**

This establishes eight digital health metrics as valid and robust endpoints for cross‐sectional studies and five metrics as potentially sensitive endpoints for longitudinal studies in ARSACS, thereby promising novel insights into upper limb sensorimotor control.

## Introduction

Autosomal recessive spastic ataxia of Charlevoix‐Saguenay (ARSACS) is a genetic disorder with pyramidal, cerebellar, and neuropathic impairments, all contributing to upper limb incoordination.^1^, ^2^ ARSACS is the second most frequent recessive ataxia and currently serves as a disease model for a large international natural history study for recessive ataxias.^1^ In order to personalize interventions to the specific impairment profile of an individual and to evaluate their effectiveness, sensitive and fine‐grained outcome measures of upper limb coordination are of vital importance.

However, the clinically available outcome measures for upper limb coordination in persons with ARSACS are either based on a subjective and crude rating of movement quality or the time to complete functional tasks.^3^, ^4^ Hence, such outcomes are not optimally suited to detect fine differences between individuals or to be sensitive to change in a clinical or research context. In addition, these assessments are not able to reveal the contribution of specific sensorimotor impairments, associated with the pyramidal, cerebellar, or neuropathic system, to abnormal performance in goal‐directed tasks. Thus, in order to personalize interventions to the specific impairment profile of an individual and to evaluate their effectiveness, sensitive, and fine‐grained outcome measures are of vital importance.

Technology‐aided assessments can record objective data about upper limb movement patterns and grip forces during goal‐directed functional tasks.^5^, ^6^ These traces are expected to reveal interindividual differences in task performance that relate to the specific sensorimotor impairment profile of an individual. In addition, these traces can be transformed into digital health metrics, describing for example movement smoothness or efficiency. These can serve as complementary, objective quantitative outcomes that promise to be more sensitive to change, thereby potentially allowing for a reduction of required sample sizes for clinical trials.^7^ Technology‐aided assessments have been successfully validated and applied in the stroke population,^7^, ^8^, ^9^ but only found little attention in recessive ataxias.^10^, ^11^ Most importantly, before using digital health metrics as endpoints for clinical trials, it is essential to document the metrological properties for a specific target population.^11^, ^12^ Also, the dependency of these metrological properties on the number of assessment task repetitions should be investigated, as rapidly applicable assessments are required to ensure their clinical implementation.^13^, ^14^

One of such technology‐aided assessments is the Virtual Peg Insertion Test (VPIT), which features a goal‐directed pick‐and‐place task on a personal computer, relying on a haptic end‐effector with a grip force‐sensing handle.^15^ The VPIT protocol consists of an initial familiarization period and five task repetitions, which can typically be performed in about 16 min in persons with neurological injuries.^16^ Previously, a core set of 10 digital health metrics describing movement patterns and grip force control was established and validated in able‐bodied and stroke populations.^11^, ^17^ Also, as part of a pilot project, the feasibility of the VPIT in ARSACS has been successfully established.^10^

Building on this foundation, the aim of this study was to document the following metrological properties of the VPIT digital health metrics in the adult ARSACS population: (1) test–retest reliability and measurement error; (2) learning effects, and (3) concurrent validity. Furthermore, the secondary objective was to evaluate the test–retest reliability and measurement error of the metrics when considering only three instead of five task repetitions, thereby potentially further enhancing the clinical feasibility of the assessment. Based on our previous work with the VPIT in post‐stroke individuals and related literature,^5^, ^13^, ^17^ we hypothesized that a subset of the VPIT core metrics is statistically robust in ARSACS and that a reduction from five to three task repetitions leads to an acceptable decrease in robustness of the metrics.

This project will permit to provide evidence about the validity and reliability of novel digital health metrics extracted from a technology‐aided assessment in the ARSACS population, thereby promising novel insights into the mechanism of sensorimotor impairments in recessive ataxias.

## Methods

### Study design

Methodological study embedded in an observational study with a cross‐sectional and test–retest component.

### Participants and procedures

Participant recruitment was done among the registry of the neuromuscular clinic (*n* = 168) of the *Centre Intégré Universitaire de Santé et de Services Sociaux* (CIUSSS) *du Saguenay–Lac‐St‐Jean* (Québec, Canada). Inclusion criteria were (1) ≥16 years old, (2) genetically confirmed ARSACS diagnosis, and (3) ability to provide informed consent. A stratified random sampling strategy was used by age group (16–29, 30–39, 40–49, and 50–59 years) and sex (men and women). Patients with other diseases causing functional limitations, having a baclofen pump that might influence lower limb function, or being pregnant were excluded. The study was approved by the ethics review board of the *CIUSSS* Saguenay–Lac‐St‐Jean (ID MP‐04‐2016‐166) and written informed consent was obtained from participants.

**Figure 1:**
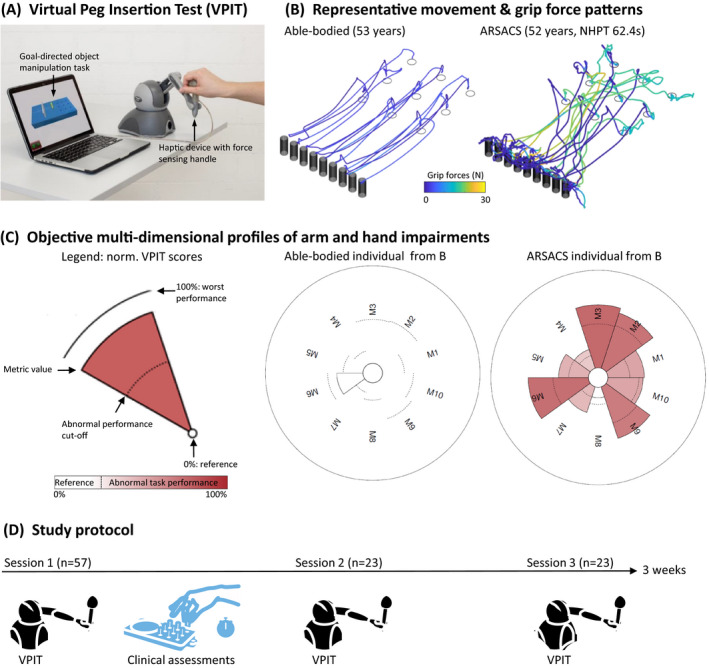
Overview of the Virtual Peg Insertion Test (A), the collected kinematic and kinetic data (B), the processed impairment profiles relying on 10 sensor‐based digital health metrics (C, M1–10) data, as well as the study protocol (D). In panel (B), the able‐bodied participant is a 53‐year‐old female, whereas the ARSACS participant is a 52‐year‐old male with a NHPT score of 62.4 sec. In the middle and right part of panel (C), each pie segment represents the value of one sensor‐based digital health metric extracted from the VPIT data of the participants in panel (B). The left part of panel (C) provides the legend for the visualization. In more details, the metrics are normalized on the range ]−∞, +∞[, with 0% (inner radius of the circle) indicating the median of an able‐bodied reference population and 100% (outer radius of the circle) the worst neurological subject in the VPIT database. For visualization, values below 0% (i.e., better than the reference population) are not displayed. The dashed circular line indicates the 95th percentile of the reference population, which is used to define abnormal task performance. ARSACS, Autosomal Recessive Spastic Ataxia of Charlevoix‐Saguenay; NHPT, Nine Hole Peg Test; M1‐M10: M1, log jerk transport; M2, log jerk return; M3, SAL return; M4, path length ratio transport; M5, path length ratio return; M6, velocity max return; M7, jerk peg approach; M8, force peaks transport; M9, force rate SAL transport; M10, force rate SAL hole approach; SAL, spectral arc length; VPIT, Virtual Peg Insertion Test. [Colour figure can be viewed at wileyonlinelibrary.com]

Participants performed the VPIT protocol and a battery of clinical assessments in a standardized order (Fig. 1D), which was used to study concurrent validity (*validity dataset*). Furthermore, a subset of participants took part in the reliability study where the VPIT was administered again within a period of 3 weeks to study test–retest reliability (*reliability dataset*).

### Technology‐aided assessment: VPIT

The VPIT (Fig. 1A) is a technology‐aided assessments for quantitatively assessing upper limb movement patterns and grip force control.^15^ The approach consisting of a commercially available, CE‐certified haptic end‐effector (PhantomOmni or Touch, 3D Systems, Rock Hill, SC, US), a custom‐made force sensing handle with piezoresistive sensors (CentoNewton, PEWATRON, Zurich, Switzerland), and a virtual reality environment displaying a goal‐directed task on a personal computer. In more details, the task displays nine virtual pegs that need to be transported into nine virtual holes by coordinating arm movements and handgrip forces. Specifically, a peg can be picked up by first aligning a virtual cursor, controlled by the haptic end‐effector, with the peg and then applying and maintaining a grip force of at least 2N. Subsequently, the peg can be transported towards the hole and inserted in it by reducing the grip force below 2N. Throughout the task, the virtual pegboard is haptically rendered by the end‐effector device and a timer displays the elapsed time. Before performing the task, participants received standardized instructions and are seated in a standardized position with approximately 45° shoulder abduction, 10° shoulder flexion, and 90° elbow flexion. The standard VPIT protocol consists of an initial familiarization period, where participants can understand how to use the haptic end‐effector, and subsequently five repetitions of the task that are used for post‐processing.

This highly standardized setup allows to gather high quality data on end‐effector position and hand grip forces at a sampling rate of 1 kHz.^15^ These data are fed into a previously established state of the art signal processing pipeline, allowing to extract 10 sensor‐based core metrics describing movement smoothness, speed, accuracy, efficiency, and grip force control during different behavioral phases of the task.^11^ These are defined as the *transport* phase (gross movement from peg pickup until insertion), the *return* phase (gross movement from peg insertion until next peg pickup), the *peg approach* phase (fine movement before picking up a peg), and the *hole approach* (fine movement before inserting a peg). A detailed pathophysiological motivation and mathematical definition of the 10 sensor‐based metrics, which were selected based on a data‐driven statistical procedure from a set of 77 candidate metrics, is provided in previous work^11^ and is briefly summarized in the following: Movement smoothness is typically expressed through bell‐shaped velocity profiles of a goal‐directed movement in able‐bodied controls and deviations from the typical bell‐shape are observed in neurological disorders.^18^, ^19^ Herein, smoothness was characterized using the normalized logarithmic jerk metric calculated during transport and return (*log jerk transport/return*), as well as the spectral arc length metric calculated during return (*SPARC return*).^18^, ^19^ Movement efficiency is typically expressed through straight movements between start and target in able‐bodied controls, whereas neurological subjects often perform uncoordinated movements with inefficient, curved trajectories and exhibit a spatial overshoot when approaching the target, which is a hallmark symptom in persons with cerebellar disorders.^20^, ^21^, ^22^ In this work, we relied on the *path length ratio* (ratio of shortest possible distance divided by actually covered distance) calculated during *transport* and *return* to describe movement efficiency.^20^ In addition, neurological disorders can affect movement speed, which was characterized using the maximum velocity during the *return* phase (*velocity max. return*). Also, potentially altered endpoint precision was described using a jerk‐based metric calculated during the *peg approach* phase (*jerk peg approach)*.^11^ Moreover, three metrics were defined to describe the smoothness of grip force coordination, namely the number of peaks in the grip force rate profile during the *transport* phase (*grip force rate num. peaks transport*) as well as the spectral arc length of the grip force rate profile during *transport* (*grip force rate SPARC transport)* and *hole approach (grip force rate SAPRC hole approach)*. After the calculation of the metrics, the effect of demographic factors, such as age and gender, is removed through mixed effect models and the metrics are normalized with respect to a normative population of 120 able‐bodied controls as well as previously recorded neurological subjects.^11^ This provides a standardized representation of the metrics on an unbounded, continuous scale from ]−∞, +∞[, where 0% corresponds to the median performance of the normative population, 100% to worst recorded task performance, and values below 0% to superior performance than the median of the normative population.

These core metrics were initially selected because of their excellent metrological properties in able‐bodied controls and their ability to discriminative a normative reference population and a population of 89 persons with neurological disorders.^11^ Furthermore, we showed that the 10 core metrics are valid to describe sensorimotor impairments in stroke populations and that three metrics, namely the *log jerk transport, log jerk return,* and *force rate SPARC transport*, are expected to provide sensitive outcomes for monitoring physiological changes in longitudinal studies with stroke populations.^17^

### Clinical assessments

A battery of clinical assessments was performed to capture different sensorimotor impairments and activity limitations.

The severity of cerebellar ataxia symptoms was assessed using the Scale for the Assessment and Rating of Ataxia (SARA), which rates typical ataxic features including gait, upper limb deficits, and speech on a scale from 0 (no ataxia) to 40 (most severe ataxia).^3^ Power grip strength was measured using a Jamar dynamometer with a standardized procedure and three repetitions, with the average being used as the outcome measure.^23^ Lateral pinch strength between the thumb and index was measured using a pinch gauge and three repetitions (Baseline Pinch Gauge, Fabrication Enterprises Inc., Irvington, NY). Fine dexterity was assessed using the Nine Hole Peg Test (NHPT), which requires participants to insert and then to transport nine pegs in nine holes as quickly as possible.^4^ The total time to complete the task is recorded in seconds and the average of two trials was used for analyses. Upper limb coordination was assessed using the standardized finger‐nose test (SFNT).^24^ The SFNT requires participants to perform as many reaching movements as possible within 20 sec, alternating between a 40 cm distant target and the participants' nose. Lower limb motor coordination was assessed with the Lower Extremity Motor Coordination Test (LEMOCOT).^25^ Seated on a regular chair, participants alternately touched two targets placed 30 cm apart on the floor with their foot. One trial was performed for each side and the number of targets touched in 20 sec was recorded. The LEMOCOT was found to be valid and reliable in the adult ARSACS population.^25^ Functional independence in activities of daily living was measured using the Barthel Index for Activities of Daily Living (French version). Ten items were rated, for a maximum score of 100 representing total independence.^26^

### Data analysis

In order to characterize the metrological properties of the VPIT metrics, the COSMIN framework was used,^12^ and adapted for digital health metrics.^11^, ^17^ Descriptive statistics were used for continuous variables (median, interquartile, ranges), and frequency and percentage for categorical variables.

To describe test–retest reliability, we calculated the intraclass correlation coefficient (ICC, version A,k) across all three measurement sessions. The ICC considers the intra‐ and interparticipant variability of a metric and describes its ability to discriminate multiple participants, with an ICC > 0.7 being defined as excellent test–retest reliability.^27^ Moreover, we reported the smallest real difference (SRD) of a metric, which is the range of values where it is not possible to distinguish between measurement noise and an actual change in the measured construct, which is especially important for sensitively capturing longitudinal changes.^28^ The SRD was calculated as $1.96\times \sqrt{2}\times \sqrt{1-\mathrm{ICC}}$ and normalized with respect to the range of observed values, such that it can be compared across metrics, leading to the cut‐off of SRD% ≤30.3 to identify metrics with relatively small measurement error.^11^ To check for systematic bias in the metrics across assessment timepoints, a Bland–Altman analysis was performed.^29^ Also, the normalized slope (*η*) across assessment timepoints was calculated to describe potential systematic learning effects, which might mask physiological changes observed in longitudinal studies. Strong learning effects were present if *η* was below −6.35% and statistically significant according to a paired *t*‐test.^11^ These analyses were performed on the *reliability* dataset, containing three repeated measurement sessions, each with five repetitions of the VPIT task. To further evaluate if the number of task repetitions could be reduced to enhance clinical feasibility, we repeated the described analysis when only considering the first three repetitions of the task at each timepoint.

For analyzing concurrent validity, we relied on the *validity* dataset, containing data from a single assessment session with three VPIT repetitions and performed a Spearman correlation analysis between digital health metrics and the clinical outcome measures. We defined a priori hypotheses about the expected correlations based on the physiological motivation of the metrics and clinical scales, and previous studies in neurological disorders.^5^, ^11^, ^17^ It is essential to mention that digital health metrics are expected to provide complementary information to the clinical scales, therefore typically leading to low to moderate correlations (e.g., correlation coefficients between 0.3 and 0.7).^5^, ^30^ In more details, we expected digital health metrics describing arm movement control to moderately correlate with clinical assessments involving arm movements, namely the SARA, its composite score focusing on the upper limb (summation of results from SARA‐alternating hand movements, SARA‐finger to nose test, and SARA‐finger chase test), the NHPT and the SFNT. Similarly, we expected moderate correlations between digital health metrics describing the precise control of hand grip forces to clinical assessments requiring precise grip force control, namely the NHPT. Furthermore, we expected low correlations between the digital health metrics and clinical assessments of grip and pinch strength, as it is assumed that force control and strength are different, separable physiological systems.^31^, ^32^ In addition, we expected low correlations with the LEMOCOT, as it is a measure of lower limb coordination, thereby being separated from upper limb coordination. Lastly, we expected a low correlation between the Barthel index and the digital health metrics, as the latter describes patterns of behavioral variability in a task‐related setting that might not directly be related to independence of daily life.

## Results

A total of 57 participants (mean age: 35.0 years, 47.4% were men) were recruited in the validity study (*validity* dataset, Fig. 1, Table 1) and 23 participants (mean age: 35.0 years, 47.8% were men) were recruited in the test–retest reliability study (*reliability* dataset, Fig. 1, Table 1).

**Table 1:** Characteristics of the participants used for evaluating the reliability and validity of the VPIT metrics.

Characteristic	ARSACS validity dataset (*n* = 57, 1 session)	ARSACS reliability dataset (*n* = 23, 3 sessions)
Age, (year)	35.0 ± 13.5 (16–61)	35.0 ± 11.0 (27–57)
Sex, *n* (%)		
Men	27 (47.4)	11 (47.8)
Women	30 (52.6)	12 (52.2)
Homozygous, *n* (%)	52 (92.8) *n* = 56	23 (100)
SARA (0–40)	19 ± 14 (4–36) *n* = 56	20.5 ± 10.6 (7–36)
NHPT (sec)	44.2 ± 23.5 (23.9–144.9) *n* = 56	45.3 ± 18.4 (23.9–105.5)
SFNT (# of repetitions)	10.4 ± 4 (5.8–21.3) *n* = 56	10.5 ± 4.3 (6.0–21.3)
Grip strength (kg)	29.2 ± 15.9 (17.2–59.1) *n* = 55	24.7 ± 16.6 (17.2–59.1)
Pinch strength (kg)	5.7 ± 2.2 (3.3–10.3) *n* = 55	5.8 ± 1.9 (3.3–9.2) *n* = 22
LEMOCOT (# of repetitions)	19.0 ± 15.3 (1–48) *n* = 49	18.5 ± 6.0 (1–40) *n* = 18
Barthel index (0–100)	90 ± 20 (35–100) *n* = 54	85.0 ± 27.5 (45–100) *n* = 21

Values reported as median ± interquartile range (minimum‐maximum). If missing values were present, *n* denotes the number of participants without missing values. ARSACS, Autosomal Recessive Spastic Ataxia of Charlevoix‐Saguenay; LEMOCOT, Lower Extremity Motor Coordination Test; NHPT, Nine Hole Peg Test; SFNT, Standardized Finger‐Nose Test; SARA, Scale for the Assessment and Rating of Ataxia; VPIT, Virtual Peg Insertion Test.

### Test–retest reliability, measurement error, and learning effects

**Figure 2:**
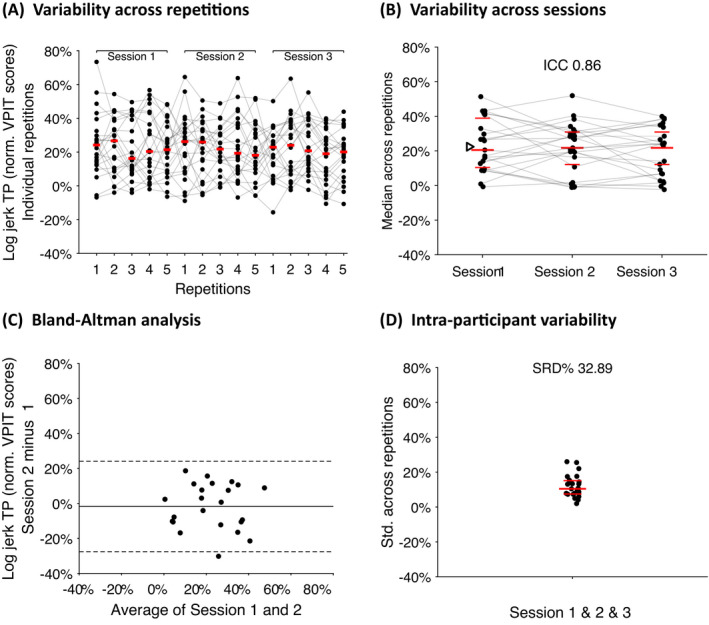
Evaluation of the VPIT metrics in ARSACS: example of the log jerk transport as an indicator of movement smoothness. The variability of the metrics across repetitions (A) and sessions (B) to investigate the presence of learning effects (slope *η* between sessions, normalized relative to range of values) and the ability of a metric to discriminate across participants (intraclass correlation coefficient, ICC). In addition, a Bland–Altman analysis (C, solid and dashed horizontal lines represent the median and 5th and 95th percentile of differences, respectively) was performed to check for systematic bias. Lastly, the intraparticipant variability and the closely related measurement error (D, smallest real difference, SRD%) of a metric were characterized. Horizontal red bars indicate 25th‐, 50th‐, and 75th‐ percentile of the characterized distribution. The triangle in panel (B) defines the 95th‐percentile of control participants, which is commonly used as a cut‐off to identify individuals with abnormal task performance. ARSACS, Autosomal Recessive Spastic Ataxia of Charlevoix‐Saguenay; Log, logarithm; TP, transport; VPIT, Virtual Peg Insertion Test. [Colour figure can be viewed at wileyonlinelibrary.com]

The evaluation of test–retest reliability (ICC), measurement error (SRD%), and learning effects (*η*) can be found in Fig. 2 and Table 2. Across metrics, the ICC was 0.87 ± 0.11 (median ± interquartile range), with only the *path length ratio return* (ICC 0.62) and the *jerk peg approach* (ICC 0.32) being below the cut‐off of 0.7. The SRD% across metrics was 30.9 ± 18.7, with the measurement error being below the cut‐off of 30.3 for five metrics, namely *log jerk return (27.5), SPARC return* (25.4), *velocity max. return* (23.8), *grip force rate num. peaks transport* (28.9), and *grip force rate SPARC transport* (19.1). No strong learning effects were found between assessment timepoint one and two (*η* −1.3 ± 2.5) and between assessment timepoint two and three (*η* −0.1 ± 2.8). Even though a statistically significant learning effect was visible between timepoint two and three for the *velocity max. return* metric (*η* −5.07, *p* = 0.019, *t* = −2.51, DoF = 22), the effect was not deemed as strong according to the cut‐off (−6.35%).

**Table 2:** Evaluation of reliability and learning effects of the VPIT metrics considering five task repetitions and three repeated assessment sessions.

Digital health metrics	Reliability (5 VPIT repetitions)		Learning effects (5 VPIT repetitions)	
	ICC [CI]	SRD%	Norm. slope *η* (session 1 & 2)	Norm. slope *η* (session 2 & 3)
Log jerk transport	**0.86** [0.81, 0.90]	32.89	**1.31**	**−0.11**
Log jerk return	**0.92** [0.89, 0.94]	**27.51**	**−2.33**	**2.97**
SPARC return	**0.93** [0.90, 0.95]	**25.42**	**0.20**	**0.14**
Path length ratio transport	**0.80** [0.73, 0.86]	44.08	**−2.36**	**3.48**
Path length ratio return	0.62 [0.48, 0.73]	45.23	**−2.95**	**−2.68**
Velocity max. Return	**0.90** [0.86, 0.93]	**23.84**	**3.25**	−5.07*
Jerk peg approach	0.33 [0.09, 0.52]	55.48	**−1.84**	**−0.09**
Grip force rate num. peaks transport	**0.82** [0.76, 0.87]	**28.92**	**0.00**	**0.00**
Grip force rate SPARC transport	**0.91** [0.88, 0.94]	**19.14**	**−1.42**	**−0.14**
Grip force rate SPARC hole approach	**0.88** [0.83, 0.91]	34.55	**−1.25**	**−4.29**

ICC, intraclass correlation; CI, confidence interval; SRD%, smallest real difference; VPIT, Virtual Peg Insertion Test.

*p < 0.05, **p < 0.001 for paired t‐test between sessions. For all three statistics, accepted cut‐offs (ICC > 0.7, SRD% <30.3, *η* > −6.35 or non‐significant) were used to determine if a metric fulfills each of the evaluation criteria (values in bold font).

When only considering three instead of five repetitions of the VPIT (Table S1), across metrics, the ICC reduced by 0.04 ± 0.06 (min 0.01, max 0.30 for *jerk peg approach*), the SRD% increased by 1.72 ± 3.75 (min 0.05, max 32.27 for *jerk peg approach*), *η* between timepoint one and two increased by 0.02 ± 1.90 (min 0, max 6.28 for *log jerk return*), and *η* between timepoint two and three increased by 1.08 ± 5.84 (min 0, max 7.04 for *log jerk return)*. When only considering three instead of five repetitions, the same eight metrics were reliable, two additional metrics had insufficient measurement error (*log jerk return 31.3* and *grip force rate num. peaks transport 31.7)*, and no metrics showed strong learning effects.

### Concurrent validity

The hypothesis related to the expected correlations with the NHPT was partially fulfilled, as significant moderate correlations were found with metrics describing movement control (*SPARC return ρ* = −0.32, *p* = 0.017 and *velocity max. return ρ* = 0.46, *p* = 0.0004), but no significant correlations were observed with metrics describing grip force control. The hypothesis related to the expected correlations with the SFNT was fulfilled, as significant moderate correlations were observed with metrics describing movement control (*SPARC return ρ* = −0.39, *p* = 0.003 and *velocity max. return ρ* = −0.51, *p* < 0.0001). The hypothesis related to the expected correlations with grip strength was fulfilled, as no significant correlations were observed. The hypothesis related to the expected correlations with pinch strength was partially fulfilled, as no significant correlations with metrics of grip force control were observed, but instead a significant correlation with a metric of arm control (*velocity max. return ρ* = −0.39, *p* = 0.0039). The hypothesis related to the expected correlations with the LEMOCOT was not fulfilled, as significant correlations were observed with metrics of arm control (*velocity max. return ρ* = −0.32, *p* = 0.021) and grip force control (*grip force rate num. peaks transport ρ* = −0.53, *p* < 0.0001 and *grip force rate SPARC transport ρ* = −0.39, *p* = 0.0044). The hypothesis related to the expected correlations with the Barthel index was fulfilled, as no significant correlations were observed. For the SARA, the hypotheses were fulfilled, as significant correlations of metrics describing arm movements were observed with the overall SARA (*velocity max. return ρ* = 0.32, *p* < 0.05) and its composite upper limb score (*path length ratio transport ρ* = 0.34, *p* < 0.05, *path length ratio return ρ* = 0.33, *p* < 0.05 and *velocity max return* (*ρ* = 0.29, *p* < 0.05). Thus, overall, four hypotheses related to the concurrent validity of the VPIT metrics were fulfilled, two partially fulfilled, and one not fulfilled (Table 3).

**Table 3:** Concurrent validity (Spearman correlations) between VPIT digital health metrics and clinical assessments.

Digital health metrics	Clinical assessments						
	Nine Hole Peg Test	Standardized finger to nose test	Grip strength	Pinch strength	Lower extremity motor coordination test	Barthel index	Scale for the assessment and rating of ataxia – upper limb
Log jerk transport	0.15	−0.18	−0.23	−0.15	−0.01	−0.08	0.11
Log jerk return	0.20	−0.11	−0.12	−0.01	0.16	0.11	−0.01
SPARC return	**−0.32** *	**−0.39** *	−0.24	−0.1	0.01	−0.1	0.25
Path length ratio transport	0.25	−0.16	−0.04	0.00	0.05	−0.12	**0.34** *
Path length ratio return	0.17	−0.13	−0.27	−0.01	0.16	0.00	**0.33** *
Velocity max. return	**0.46** **	**−0.51** **	−0.21	**−0.39** *	**−0.32** *	−0.24	**0.29** *
Jerk peg approach	0.10	0.04	−0.15	−0.1	0.09	0.09	0.08
Grip force rate num. peaks transport	0.21	−0.18	−0.13	−0.21	**−0.53** **	0.00	0.06
Grip force rate SPARC transport	0.26	−0.24	0.04	−0.16	**−0.39** *	−0.15	0.17
Grip force rate SPARC hole approach	0.21	−0.06	−0.12	−0.10	−0.14	−0.09	0.18
	Hypothesis partially fulfilled	Hypothesis fulfilled	Hypothesis fulfilled	Hypothesis partially fulfilled	Hypothesis not fulfilled	Hypothesis fulfilled	Hypothesis fulfilled

Bold font indicates statistically significant correlations. SPARC, spectral arc length.

^*^ *p* < 0.05.

^**^ *p* < 0.001.

## Discussion

The aim of this work was to document metrological properties of a previously established core set of 10 digital health metrics extracted from the VPIT, a technology‐aided assessment of upper limb movement patterns and grip force control, and ensure that those can be used as novel, insightful clinical endpoints for studies in persons with ARSACS.

The need for novel, sensitive, and fine‐grained assessments of upper limb coordination in ARSACS is exemplified by a longitudinal study that relied on clinical scales and observed that, over a 2‐year period, lower limb coordination, balance, walking abilities, and overall disease severity, but not upper limb coordination, deteriorated significantly.^35^ This is surprising, given that it is expected that the significant deterioration in these body functions reflect pathological changes in the pyramidal and cerebellar systems that should also impact upper limb coordination. Hence, it was speculated that the absence of significant deterioration in upper limb coordination was mainly an artifact of the available outcome measures.^35^ These included the NHPT, which seems to not be sensitive enough to detect fine alterations in motor control, especially in persons that show considerable impairments in upper limb distal strength.^35^

### Eight VPIT metrics are well‐suited for cross‐sectional studies in persons with ARSACS

We found that eight of the VPIT core metrics have excellent test–retest reliability and five have relatively low measurement error across three assessment sessions. In addition, none of the metrics has strong learning effects across sessions, with only a slight improvement in speed visible between sessions two and three that was below the established cut‐off. This provides evidence that eight VPIT metrics have robust statistical properties to accurately and objectively characterize sensorimotor impairments in cross‐sectional studies in persons with ARSACS. Furthermore, this establishes the foundation for the integration of five VPIT metrics as potentially sensitive endpoints in longitudinal studies. In the future, the responsiveness of the VPIT metrics should be fully evaluated, for example by capturing longitudinal changes in sensorimotor impairments and comparing them to the herein established SRD values.^36^ The relatively high measurement error in certain metrics likely results from the ability of technology‐aided to capture behavioral variability in task execution, which would have not been covered by clinical scales.^17^

Furthermore, we observed that a reduction of the number of repetitions in the assessment protocol can be achieved without large changes in the metrological properties of the metrics, except for the *jerk approach peg* metric, which was anyways deemed as not reliable in the first place. However, slight changes in the measurement error of two metrics led them to be slightly above the defined cut‐off. Given that the measurement error is mainly relevant when longitudinally assessing sensorimotor impairments, this does not affect the applicability of the metrics for cross‐sectional studies. Thus, these results further increase the clinical feasibility of the VPIT, making it applicable in approximately 10 min per body side, which comes at an acceptable reduction in the robustness of the VPIT metrics that are relevant in ARSACS. This is an important result, as time constraints have been identified as one of the key factors hindering the clinical integration of technology‐aided assessments.^14^

For the test–retest reliability analysis, the obtained metrological properties are in general in line with the values reported in literature.^5^, ^30^ Interestingly, in the previous evaluation of the VPIT metrics in post‐stroke population, four out of the 10 core metrics showed considerable learning effects across sessions.^17^ On the contrary, no strong learning effects were observed in persons with ARSACS, suggesting that they could successfully learn the task already within the first training and repetitions of the task. Reasons for this might be that post‐stroke population are typically of older age and have stronger cognitive impairment than ARSACS population.^37^ This highlights the importance of evaluating the sensor‐based metric separately for each envisioned target population, which is rarely implemented for infrequent diseases such as ARSACS.

Interestingly, the metrics *jerk peg approach* and *path length ratio return* had suboptimal reliability and measurement error values, which has also been observed in chronic post‐stroke performing the VPIT (Kanzler et al. 2020). Given that similar metrics reported during other phases of the task had excellent statistical properties (e.g., *path length ratio transport* or *log jerk transport*), it is likely that task‐related variability leads to these results and not the mathematical definition of the metrics per se. This emphasizes that it is challenging to recommend specific metrics that are statistically valid for all technology‐aided assessments and that instead data‐driven selection approaches should be leveraged to define a core set of robust metrics for each task.^11^

### The VPIT metrics are valid descriptors of upper limb coordination in ARSACS

The analysis of concurrent validity suggests that three of the initially formed hypotheses were fulfilled (SFNT, grip strength, Barthel), two partially fulfilled (NHPT, pinch strength), and one not fulfilled (LEMOCOT). Overall, the correlations between the VPIT metrics and the clinical scales were low to moderate, which is line with previous work with the VPIT in neurological disorders.^11^, ^16^ This highlights that VPIT metrics and clinical scores are related, but not redundant, thereby emphasizing their value as complementary endpoints that can help to provide novel insights into sensorimotor impairments in ARSACS. However, it is important to mention that other studies showed stronger correlations between digital health metrics and clinical scales in persons with ataxia, for example when relying on simple reaching or pick‐and‐place tasks with an instrumented spoon, a computer mouse, or a planar robotic end‐effector.^5^, ^33^, ^34^, ^38^ The reason for this might be that these simple instrumented tasks are more similar to the tasks typically included in clinical scales. Instead, the VPIT is a more complex task and might probe slightly different behavioral constructs, including, for example, a visuomotor component that is required for learning the mapping between 3D end‐effector movements and virtual reality environment. This could be seen as a limitation of the VPIT when comparing it to standard clinical scales.

As expected, two kinematic VPIT metrics describing gross movements (SPARC return, velocity max. return) correlated moderately with the NHPT and the SFNT, thereby confirming that these metrics capture aspects of fine and gross upper limb coordination. However, we would also have expected a correlation between the kinetic VPIT metrics and the NHPT, given that both assess components of grip force control. The low correlation therein might arise from the different grip types required for the VPIT (power grip) and the NHPT (precision grip). Also, the absence of a correlation between the kinetic VPIT metrics and the NHPT might be because the NHPT only provides one compound outcome measure describing both movement and grip force control.

We expected low correlations between the digital health metrics and indicators of grip/pinch strength, given that different body systems are involved in precise grip force control (VPIT metrics) and grip strength (clinical scores).^31^, ^39^ However, a modest correlation between VPIT digital health metrics describing speed during goal‐directed movements with pinch strength was observed. This might indicate that impairments in arm movements and grip strength are similarly affected by disease severity in ARSACS.

Furthermore, we observed moderate significant correlations between three digital health metrics (movement speed during return and grip force control during transport) and the LEMOCOT, an assessment of lower limb coordination supposed to capture especially the pyramidal and cerebellar features of ARSACS.^25^, ^40^ These correlations were more consistent and pronounced than the ones observed with the clinical upper limb functional tasks, such as the NHPT and the SFNT. This could be explained by a shared physiological mechanism of disrupted lower and upper limb coordination in ARSACS. It is likely that this mechanism is driven by cerebellar and pyramidal components, given that the correlations between the VPIT metrics and grip/pinch strength, as a typical descriptor of pyramidal and neuropathic disease features, were less pronounced.^21^, ^22^, ^41^ This indicates that the VPIT metrics describe not one specific disease feature but rather upper limb coordination as a construct that is influenced especially by pyramidal and cerebellar components. Further support for this is provided by the significant correlations of certain VPIT metrics with the SARA upper limb items, which are known to capture cerebellar disease features.^3^ Also, this suggests that the impairment‐based metrics of the VPIT are indeed able to inform on suboptimal motor control, whereas the activity‐based assessment of the NHPT and SFNT capture the effect of these impairments on a functional level.

As expected, no considerable correlations between the VPIT digital health metrics and independence in daily life, as measured by the Barthel index, were found. This is likely because the included persons with ARSACS still had overall high levels of independence and the interparticipant variability was small, thereby precluding a meaningful correlation analysis (Barthel index values 90 ± 20, scale maximum at 100).

## Conclusions

This work provides evidence for the robustness and validity of eight sensor‐based digital health metrics of the VPIT as insightful endpoints that objectively characterize upper limb movement patterns and grip forces in persons with ARSACS. In addition, five of the metrics have well‐suited statistical properties to serve as potentially sensitive endpoints in longitudinal studies. Further, an adapted protocol of the VPIT with three instead of five task repetitions was successfully established, thereby further enhancing the clinical feasibility of the VPIT. This opens up novel avenues for thoroughly studying impaired upper limb coordination in ARSACS and to reconsider the temporal evolution of upper limb sensorimotor impairments in persons with ARSACS in the future.

## Acknowledgements

The authors thank all study participants. The study was funded by the Canadian Institutes of Health Research (Emerging Team Grant no TR2‐119189), Fondation de l'Ataxie Charlevoix‐Saguenay, and Muscular Dystrophy Canada. Additionally, the research was conducted as part of the Future Health Technologies programme which was established collaboratively between ETH Zurich and the National Research Foundation Singapore. This research is supported by the National Research Foundation, Prime Minister's Office, Singapore under its Campus for Research Excellence and Technological Enterprise (CREATE) programme. CG holds a career‐grant funding from Fonds de recherche du Québec‐santé (no 31011).

The authors declare that the funding bodies did not influence the design of the study, the collection, analysis, and interpretation of data, and the writing of the manuscript. Open Access funding enabled and organized by Projekt DEAL.

## Conflict of Interest

The authors declare that they have no conflict of interest.

## Supporting information


**Table S1.** Reliability and learning effects of the VPIT metrics considering 3 task repetitions.
