# Supplementary material for: Reliability and validity of digital health metrics for assessing arm and hand impairments in an ataxic disorder
Source: Ann Clin Transl Neurol. 2022 Feb 27;9(4):432–43. doi: 10.1002/acn3.51493 (PMC8994987; doi:10.1002/acn3.51493)
Supplement: Supplementary file 1 — Table S1. Reliability and learning effects of the VPIT metrics considering 3 task repetitions. [file ACN3-9-432-s001.docx]

**Supplementary materials**

**Title:** Reliability and validity of digital health metrics for assessing arm and hand impairments in an ataxic disorder

**Authors:**

Christoph M. Kanzler^1,2^, Isabelle Lessard^3^, Roger Gassert^1,2^, Bernard Brais^4^, Cynthia Gagnon^3,5^*, Olivier Lambercy^1,2^*

*authors contributed equally.

1 Rehabilitation Engineering Laboratory, Institute of Robotics and Intelligent Systems, Department of Health Sciences and Technology, ETH Zurich, Zurich, Switzerland.

2 Future Health Technologies, Singapore-ETH Centre, Campus for Research Excellence And Technological Enterprise (CREATE), Singapore.

3 Groupe de recherche interdisciplinaire sur les maladies neuromusculaires (GRIMN), Centre intégré universitaire de santé et de services sociaux du Saguenay–Lac-St-Jean, Québec, Canada.

4 The Montreal Neurological Institute and Hospital, McGill University, Québec, Canada.

5 Faculty of Medicine and Health Sciences, Université de Sherbrooke, Québec, Canada.

**Corresponding author:** Christoph M. Kanzler ([christoph.kanzler@sec.ethz.ch](mailto:christoph.kanzler@sec.ethz.ch))

**Table SM1** **Reliability and learning effects of the VPIT metrics considering 3 task repetitions**.

| **Digital health metrics** | **Reliability**  (3 VPIT repetitions) | | **Learning effects**  (3 VPIT repetitions) | |
| --- | --- | --- | --- | --- |
|  | **ICC [CI]** | **SRD%** | **Norm. slope η**  (session 1 & 2) | **Norm. slope η**  (session 2 & 3) |
| Log jerk transport | **0.82** [0.74, 0.89] | 33.64 | **2.76** | **4.28** |
| Log jerk return | **0.88** [0.81, 0.92] | 31.26 | **3.95** | **-4.07** |
| SPARC return | **0.92** [0.88, 0.95] | **26.75** | **0.17** | **-4.05** |
| Path length ratio transport | **0.74** [0.61, 0.83] | 43.58 | **-2.68** | **1.31** |
| Path length ratio return | 0.35 [0.03, 0.57] | 44.91 | **-4.57** | **-0.23** |
| Velocity max. return | **0.88** [0.82, 0.92] | **25.94** | **2.80** | **-8.46** |
| Jerk peg approach | 0.03 [-0.45, 0.37] | 87.75 | **-1.33** | **-0.04** |
| Grip force rate num. peaks transport | **0.74** [0.61, 0.83] | 31.72 | **0.00** | **0.00** |
| Grip force rate SPARC transport | **0.89** [0.84, 0.93] | **22.97** | **2.50** | **-2.70** |
| Grip force rate hole approach | **0.85** [0.78, 0.90] | 34.55 | **-3.77** | **-0.42** |

*^ICC: intra-class correlation. CI: confidence interval. SRD%: smallest real difference. *p<0.05, **p<0.001 for paired t-test between sessions. For all three statistics, accepted cut-offs (ICC >0.7, SRD% <30.3, η> -6.35 or non-significant) were used to determine if a metric fulfils each of the evaluation criteria (values in bold font).^*
